# Iron mother- protocol for a randomised controlled trial of daily versus alternate day ferrous fumarate for the treatment of iron deficiency anaemia in pregnancy

**DOI:** 10.1016/j.conctc.2025.101447

**Published:** 2025-02-03

**Authors:** F.E. O'Toole, F.M. McAuliffe, J.M. Fitzgerald, G.A. Mealy, R. Petkute, L.A. Bolger, A. Murphy-Cruse, B. Soldati, M. Galligan, J.M. Walsh

**Affiliations:** a UCD Perinatal Research Centre, University College Dublin, National Maternity Hospital, Dublin, Ireland; b National Maternity Hospital, Dublin, Ireland

**Keywords:** Iron deficiency anaemia, Pregnancy, Ferrous fumarate, Oral iron supplementation, Maternal anaemia

## Abstract

**Objective:**

Iron deficiency anaemia (IDA) is the commonest haematological problem in pregnancy and has implications for maternal, fetal, and childhood health. Treatment, despite being inexpensive and readily available, remains challenging with issues relating to compliance, tolerability, and effectiveness. There is a lack of consensus regarding the optimal dosing of oral iron replacement in pregnancy. Emerging evidence from non-pregnant populations suggest that alternate day dosing may be as effective.

**Methods:**

We propose a phase IV open label randomised controlled non-inferiority trial of daily versus alternate day ferrous fumarate for a 4-week period for the treatment of confirmed iron deficiency anaemia in pregnancy. Our study population comprises singleton pregnancies between 14+0- and 34+0-weeks’ gestation with a haemoglobin (Hb) of <10.5g/dL and a ferritin of <30μg/L. The intervention is alternate day ferrous fumarate 305mg (100mg elemental iron) and the comparator is daily ferrous fumarate 305mg. The primary endpoint, change in Hb from randomisation to week 4, will be analysed by linear regression, adjusting for baseline Hb level. Analysis will be conducted by intention-to-treat analysis with per protocol sensitivity analysis. Sample size was calculated on the assumption of no difference between primary endpoint means, a Type 1 error rate of 0.025, a power of 90 %, a standard deviation of 0.83 g/dL and a non-inferiority margin of −0.4 g/dL. Under these assumptions, 92 subjects per treatment arm would be required to test for non-inferiority.

**Conclusion:**

We hypothesise that alternate day iron in pregnancy will be as effective as daily iron for the treatment of iron deficiency anaemia.

## Highlights

• Randomised controlled trial of daily versus alternate day oral iron for the treatment of anaemia in pregnancy.
• Inclusion criteria: singletons with haemoglobin < 10.5g/dL and ferritin < 30μg/L between 14+0 and 34+0 weeks gestation
• Non-inferiority study with a non-inferior margin of -0.4g/dL haemoglobin difference between the two arms.
• Secondary outcomes include compliance, tolerance, and haemoglobin levels at term

## 1 Introduction

Iron deficiency anaemia is the commonest haematological problem in pregnancy affecting approximately 38 % of women globally [1]. The prevalence in low and middle income countries approaches 50 % [2]. Rates in Western countries are also high despite much lower rates of helminthic disease, malaria and haemoglobinopathies. Cross-sectional data from fifteen European countries suggest a prevalence of 35 % of iron deficiency anaemia in pregnancy [3]. A two centre study from the UK in 2017 demonstrated that over 45 % of women had a diagnosis of anaemia in their pregnancy [4]. A 2018 audit across 86 maternity units in the UK and Ireland found rates of 30.6 % of iron deficiency anaemia in pregnancy [5].

Iron deficiency anaemia in pregnancy is associated with adverse maternal, obstetric neonatal and childhood outcomes. From a maternal perspective, iron deficiency anaemia results in a variety of symptoms including fatigue, breathlessness, and dizziness. It has been linked to increased rates of postpartum depression, lactation failure and earlier cessation of breastfeeding [[6], [7], [8], [9]]. Reported obstetric complications include increased rates of caesarean section, labour induction, postpartum haemorrhage and postpartum anaemia [10]. From a neonatal perspective, there is an increased risk of stillbirth, lower infant birth weight, pre-term birth, lower Apgar scores at delivery and increased rates of neonatal morbidity and mortality [11]. Less widely understood and of increasing concern are the long-term neurodevelopmental, cognitive, motor and behavioural associations in offspring in the setting of maternal anaemia and iron deficiency [[12], [13], [14], [15], [16], [17]].

Correction of iron deficiency anaemia in pregnancy, while in theory straight-forward, can be complicated by issues with compliance and side-effects [18]. Treatment in the form of inexpensive ferrous iron salts which are readily available are not always well-tolerated, particularly in pregnancy where physiological changes predispose to gastrointestinal symptoms [19,20]. Clear and stream-lined advice regarding the appropriate method of ingestion to maximise absorption with oral iron has been shown to improve management [21].

Recent research has shown promising evidence of improved absorption with alternate day dosing in non-pregnant women [[22], [23], [24]]. International clinical guidelines on the management of iron deficiency anaemia in pregnancy vary in terms of recommended dosing, timing, and route of supplemental iron [25]. Alternate day oral iron versus daily oral iron in pregnancy for the treatment of confirmed iron deficiency anaemia has not yet been examined in a randomised controlled trial in pregnancy, despite suggestion of its benefits from a Cochrane review [26]. IronMother is a non-inferiority randomised controlled trial of alternate day versus daily Galfer® (ferrous fumarate) for the treatment of iron deficiency anaemia in pregnancy.

## 2 Materials and methods

### 2.1 Overview

**Fig. 1:**
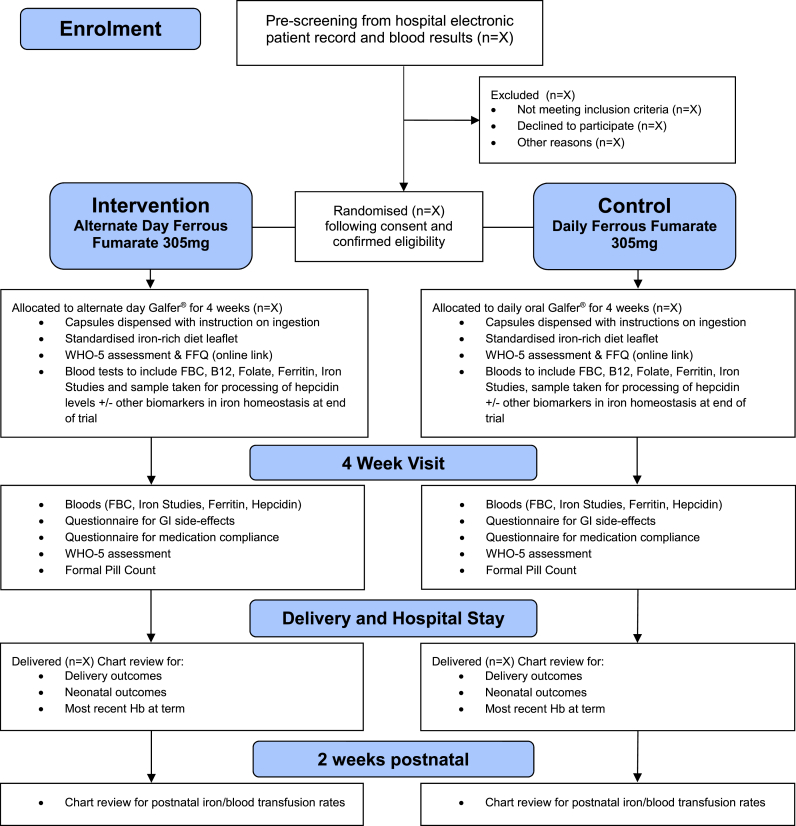
Study flow diagram- IronMother.

This protocol outlines the principles and methodology of a proposed non-inferiority randomised controlled trial of alternate day ferrous fumarate (Galfer®) compared to the standard daily ferrous fumarate for the treatment of confirmed iron deficiency anaemia in pregnancy. Fig. 1 outlines the study methodology on the basis of the principle of Standard Protocol Items: Recommendations for Interventional Trials (SPIRIT) and the Consolidated Standards of Research Trials (CONSORT). This trial is registered with The EU Clinical Trials Register (Eudra CT number: 2022-001815-25) and received approved from the Health Products Regulatory Authority in July 2022. This trial is sponsored by the Clinical Research Centre (CRC) at University College Dublin (UCD).

### 2.2 Ethics

This clinical trial received ethical approval from the National Research Ethics Committee for Clinical Trials (NREC-CT) on 6^th^ December 2022 (Application number: 22-NREC-CT-131).

### 2.3 Enrolment and eligibility

This is a single centre study at the National Maternity Hospital, Dublin, Ireland which is a large tertiary level obstetric unit with approximately 7000 deliveries a year. Candidates will be identified through follow-up of routine antenatal blood test results through the hospital antenatal out-patients department. Charts will be screened for suitability based on a low haemoglobin in pregnancy and eligible patients will be contacted by the research team to offer participation in the trial.

Women with singleton pregnancies, ≥18 years of age, between 14+0- and 34+0-weeks’ gestation and with a standard to English good enough to understand the patient-facing documentation and communicate easily with the research team will be contacted.

#### 2.3.1 Inclusion criteria

Inclusion criteria include a haemoglobin of <10.5 g/dL and a ferritin of <30 μg/L. Candidates must not already be taking therapeutic doses of supplemental iron regularly.

#### 2.3.2 Exclusion criteria

Exclusion criteria include being unable to give informed written consent, multiple pregnancy, haemoglobin <7 g/dL, current and active inflammatory disease, previous bariatric surgery, known haemoglobinopathy, known haemochromatosis and any condition which in the opinion of the investigators may put the participant at risk. Any patient recruited at ≥30 + 0 will need to have a haemoglobin of at least 8.0 g/dL or more to be eligible to participate.

Potentially eligible participants will be contacted by a trained member of the research team and offered participation and provided with both written and verbal information on the trial. Following adequate time to consider participation, a recruitment visit appointment will be arranged, where written informed consent shall be obtained by the research clinician. Study participation will be highlighted in the patient's records and a letter sent to the primary care team if randomised. Once deemed eligible to participate from the laboratory results, subject demographics shall be recorded, computerised randomisation performed, and study allocation ferrous fumarate capsules provided to the patient dispensed from the hospital pharmacy.

### 2.4 Allocation of participants

Randomisation will take place during the enrolment phase of the trial, after written informed consent is obtained from each trial subject. Each enrolled subject will be assigned a subject identification number that will uniquely identify their record in the study database. Allocation will take place according to a computer-generated variable block randomisation schedule in the ratio 1:1 across the intervention and control arms. Randomisation will be stratified by gestation (14–20 weeks, ≥20+1–28weeks, ≥28+1–34 weeks). The computer-generated randomisation schedule will be stored securely on a password protected computer. The randomisation list will be numbered using sequential 6-digit randomisation codes (e.g. IMA-001, IMA-002) – these are the codes which will uniquely identify each subject. Each arm/group assignment – daily oral iron or alternate daily iron – will be added to an allocation table with its corresponding 6- digit randomisation code. The primary researcher/investigator will obtain the allocation from REDCap (Research Electronic Data Capture), an electronic data capture system which is going to be used for this study [27]. The allocation will be obtained only at the time of randomisation and will not be accessible to the researchers or investigators prior to this point. This is an open-label study. The treatment protocol will not be blinded to investigator, subjects, or medical or nursing staff.

## 3 Sample size

Statistical analysis determined the sample size for this RCT which is 184 women. We use a fixed-margin method to choose an appropriate non-inferiority margin. As there are no trials comparing daily oral iron with placebo for the treatment of iron deficiency anaemia, we conservatively estimated that the four-week mean change in Hb with daily oral iron is 1.2 g/dL. This was based on international guidelines regarding expected rise in haemoglobins following treatment with oral iron for iron deficiency anaemia in pregnancy [[28], [29], [30]]. In order for alternate day iron to be considered non-inferior, it would need to preserve at least two thirds of this effect i.e. it would need to have a four week mean change in Hb that is not more than 0.4 g/dL less than that for daily oral iron. A non-inferiority limit of −0.4 was also specified in a previous non-inferiority trial looking at two forms of oral iron in a female population with IDA [31]. Two further non-inferiority randomised controlled trials looking at oral iron supplementation in pregnancy used non-inferiority limits of −0.3 g/dL and −0.5 g/dL and therefore our non-inferiority of 0.4 g/dL is within the range of what was considered appropriate in other studies [32,33].

Based on previously conducted trials of oral iron in pregnancy, we assumed a standard deviation of 0.83 g/dL for the 4 week change in haemoglobin levels [34,35]. Sample size calculations were conducted in the R statistical software package, using the method for non-inferiority trials outlined by Julious et al. [36] Assuming no difference between treatment groups in the mean of the primary endpoint (i.e. μ_I_ - μ_S_ = 0), a non-inferiority margin of −0.4 g/dL and standard deviation of the primary endpoint of 0.83, 92 subjects per treatment arm would be required to test for non-inferiority, in order to achieve a statistical power of 90 % with a one-sided alpha of 0.025. Anticipating a dropout rate of 20 %, approximately 230 subjects will be enrolled in the trial.

## 4 Study treatments

Eligible participants will be randomised to daily or alternate day oral ferrous fumarate (Galfer®) for a period of four weeks. This preparation of iron contains 305 mg of ferrous fumarate which is the equivalent of 100 mg elemental iron. Patients will be given specific and clear instructions regarding the optimal method of ingestion for oral iron to maximise its absorption which will include to take in the morning, on an empty stomach, if possible, approximately 1 h before their breakfast, with a source of vitamin C and not to be taken with foodstuff, medication and other nutritional supplements that may hinder absorption. The capsules for the 4 weeks will be dispensed by our hospital pharmacy and the patient requested to return all packaging and any unused capsules at the follow-up visit.

After randomisation, data will be entered into REDCap on demographics, current medications, supplements, medical and surgical history, wellbeing, and diet. Participants will be provided with the hospital standard information leaflet on how to increase dietary iron.

### 4.1 Follow-up appointment

All participants will be reviewed again after 28 days of treatment. Bloods will be taken at this visit to include a full blood count, a reticulocyte count, iron studies and ferritin. Compliance will be assessed by both a formal capsule count and compliance questionnaires. Further data will be obtained on side-effects, tolerance, and ability to comply with dosing regimen via participant questionnaires. A wellbeing survey will be repeated (WHO-5 wellbeing questionnaire). Maternal, delivery and neonatal outcomes will be obtained for each participant two weeks after delivery.

## 5 Outcome measures

### 5.1 Primary outcome

The primary objective is to investigate whether alternate day Galfer® (305 mg ferrous fumarate) is non-inferior compared to daily Galfer® (305 mg ferrous fumarate) at treating iron deficiency anaemia in pregnancy. A non-inferiority trial aims to demonstrate that the tested product is not worse than the comparator by a prespecified small margin. This amount is known as the non-inferiority margin. The margin must be based on both clinical and statistical reasoning and regulators recommend that it be based on historical evidence of the active comparator, preferably from randomised trial evidence. The margin is defined either based on the pooled estimate or based on the limit of the confidence interval (CI) that is the closest to the null effect. The fixed‐margin method, which is recommended by the Food and Drug Administration, was used in IronMother, and is conservatively defined based on the lower limit of the CI of the pooled point estimate that is closest to the null effect [37].

The primary endpoint for IronMother is the change in haemoglobin in g/dL from randomisation to four weeks, calculated as Hb_W4_ – Hb_baseline_. Then the treatment effect can be measured by μ_I_ – μ_C_, the difference between the intervention (alternative day iron) and comparator treatment (daily oral iron) in the average change from randomisation to four weeks in Haemoglobin. Haemoglobin level will be measured and compared between the two groups.

### 5.2 Secondary outcomes

The pre-specified secondary outcomes in IronMother are.

i. Compliance as measured by validated questionnaire at the 4-week assessment [38].
ii. Tolerance as measured by a validated gastrointestinal symptom questionnaire at the 4-week assessment [39].
iii. Compliance as measured by objective pill count at 4-week assessment
iv. Serum ferritin levels in both groups after 4 weeks of treatment
v. Obstetric and neonatal outcomes:
  • Delivery outcomes (livebirth, stillbirth, neonatal death)
  • Mode of delivery (vaginal delivery, Caesarean)
  • Rates and quantitation of primary postpartum haemorrhage
  • Requirement of a blood transfusion in the two weeks post-delivery
  • Requirement of a postnatal iron infusion in the two weeks post-delivery
  • The requirement for any antenatal iron transfusion prior to delivery
  • Neonatal Outcomes (neonatal high dependency unit or intensive care unit admission, neonatal Apgar scores, Birth weight)
  • Most recent haemoglobin levels prior to delivery

Exploratory endpoints include measurements such as dietary assessment at trial entry (Food Frequency questionnaire- FFQ), a record of any concomitant medication and/or nutritional supplements and mental wellbeing (WHO-5 wellbeing score) assessment will be also performed at recruitment and the follow-up visit. Blood samples will be taken and stored at baseline and at the follow-up visit 4 weeks' later for processing for other biomarkers of iron metabolism.

## 6 Trial governance and safety - adverse events (AE) and serious adverse events (SAE)

Both AEs and SAEs will be prospectively recorded for all participants. The drug trial of alternate day iron will only be for a period of 4 weeks and as such, adverse events will only be recorded in this period as following this the participants will revert to routine antenatal care. The investigator will report all serious adverse events immediately to the sponsor except for those that the protocol or investigator's brochure identifies as not requiring immediate reporting. The immediate report will be followed by detailed, written reports. The immediate and follow-up reports will identify subjects by unique code numbers assigned to the latter. The sponsor will report all SUSARs (suspected unexpected serious adverse reaction) to the Health Products Regulatory Authority and the ethics committees concerned.

The trial steering committee will meet quarterly and annual review meeting will be held with UCD Clinical Research Centre with reports provided to the Health Product Regulatory Authority (HPRA) regarding progress, dissemination, and engagement activities in previous 12-month period, and annual development safety update reports will be provided to the NREC by the sponsors. The trial management committee will meet twice a month to review study recruitment and study progress.

Given the established safety of Galfer® in pregnancy to treat iron deficiency anaemia, the fact that it is widely advised in pregnancy, and that the recently updated UK guidelines on the management of iron deficiency anaemia in pregnancy recommend alternate dosing to combat side-effects [40], this trial does not meet the requirement for a Data Safety Monitoring Board as per the EMA's guidance [41]. This is a clinical study of a non-critical indication where the patients are treated for a relatively short time and the drug under investigation is well characterised and known for not harming patients.

## 7 Statistical analysis

The primary endpoint, change in haemoglobin from randomisation to week 4, will be analysed by linear regression, adjusting for baseline Hb level and gestation at baseline. Non-inferiority will be evaluated by comparing the lower limit of the 95 % confidence interval for the treatment effect to the non-inferiority margin of −0.4 g/dL. The primary efficacy analysis will be carried out following the intention-to-treat principle, on the Full Analysis Set which is defined as all randomised subjects who received any amount of study drug. A per protocol sensitivity analysis of the primary endpoint will also be conducted on the Per Protocol (PP) set, excluding subjects who were less compliant with their assigned treatment or who switched treatments during the trial.

Intervention effects on numeric secondary endpoints will be estimated using the Full Analysis Set as a between-group difference in means with a 95 % confidence interval, adjusted for gestation at baseline and baseline value (where relevant), using linear regression or generalized linear regression model depending on data distribution. Differences between treatment groups in the patterns of change over time in Hb and ferritin levels will also be explored using linear mixed effects models, with data transformation where necessary. Intervention effects on categorical secondary endpoints will be estimated as a between-group difference in proportions with a 95 % confidence interval, and chi-squared tests will be conducted to compare proportions between groups.

Safety data will be summarized descriptively using the safety set, based on treatment actually received. No interim analysis will be performed for this trial. The statistical analysis plan will be finalized prior to database lock, with any deviations from the original plan documented.

## 8 Discussion

The purpose of this randomised controlled trial is to determine if alternate day ferrous fumarate is as effective as daily ferrous fumarate for the treatment of confirmed iron deficiency anaemia in pregnancy. In light of recent compelling scientific evidence of the advantages of alternate day oral iron, it is timely to examine the benefit in pregnancy. This study is novel in assessment of both dosing regimens in an Irish pregnancy population with confirmed iron deficiency anaemia. We hypothesise that alternate day iron will be as effective as daily iron but with a better side-effect profile.

## CRediT authorship contribution statement

**F.E. O'Toole:** Writing – review & editing, Writing – original draft, Visualization, Resources, Project administration, Methodology, Investigation, Data curation, Conceptualization. **F.M. McAuliffe:** Writing – review & editing, Visualization, Supervision, Resources, Project administration, Methodology, Investigation, Data curation, Conceptualization. **J.M. Fitzgerald:** Visualization, Supervision, Project administration, Methodology, Investigation. **G.A. Mealy:** Writing – review & editing, Visualization, Resources, Project administration. **R. Petkute:** Resources, Data curation. **L.A. Bolger:** Resources, Project administration, Methodology. **A. Murphy-Cruse:** Visualization, Resources, Project administration. **B. Soldati:** Visualization, Resources, Project administration, Methodology. **M. Galligan:** Writing – review & editing, Visualization, Validation, Resources, Methodology, Investigation. **J.M. Walsh:** Writing – review & editing, Writing – original draft, Visualization, Validation, Supervision, Resources, Project administration, Methodology, Investigation, Conceptualization.

## Declaration of competing interest

The authors declare that they have no known competing financial interests or personal relationships that could have appeared to influence the work reported in this paper.
